# Employee risk recognition and reporting of malicious elicitations: longitudinal improvement with new skills-based training

**DOI:** 10.3389/fpsyg.2024.1410426

**Published:** 2024-07-31

**Authors:** Deanna D. Caputo, Lura Danley, Nathaniel J. Ratcliff

**Affiliations:** The MITRE Corporation, McLean, VA, United States

**Keywords:** security, insider threat, insider risk, malicious elicitation, skills-based training, employee training, risk recognition, risk reporting

## Abstract

Numerous security domains would benefit from improved employee risk recognition and reporting through effective security training. This study assesses the effectiveness of a new skills-based training approach to improve risk recognition and reporting of malicious elicitations. Malicious elicitations are techniques that strategically use conversation (i.e., online, in writing, in person, or over the phone) with the sole purpose of collecting sensitive, non-publicly available information about business operations, people, or technological assets without raising suspicion. To an untrained observer, a skilled elicitor can make conversations seem analogous to many professional networking situations such as those experienced over email and at conferences. A 12-month longitudinal experimental study was conducted to test training effectiveness on employees of a large corporation that focuses on serving national security needs and the public interest. Half of participants were randomly assigned to receive traditional awareness-based training (i.e., reviewing informational slides) while the other half of participants received a new skills-based training that allowed them—over the course of five weeks—to iteratively practice skills learned in the training and receive feedback on their performance in their day-to-day work environment. Following training for both experimental groups, malicious elicitations and benign professional networking test messages were sent (via email & text message) to unaware employee participants for 12 months. Findings revealed that skills-based training improved reporting of malicious elicitations and lasted for up to 12 months compared to traditional awareness-based training.

## 1 Introduction

Security training is an essential tool for organizations to mitigate risks to their information, operations, personnel, and the physical security environments. In order to effectively mitigate risks, members of organizations must possess the knowledge and skills to recognize risk and report them to security officials through appropriate reporting channels in a timely manner. Risks are defined as threats that can cause significant operational, reputational, and financial harm. Accordingly, risk behaviors are experiences of potential threats that need to be reported which can occur in-person or virtually over the Internet (e.g., email, social media). Training is the preferred method to aid employees in improving risk recognition and reporting of security risks. However, creating and implementing effective training against potential security risks (e.g., spear phishing) can be particularly difficult to implement in workplace settings (see Caputo et al., 2014). The aim of this applied research was to develop and assess the effectiveness of a new skills-based training approach for employees to recognize and report malicious elicitation risks compared to traditional, awareness-based training.

### 1.1 Awareness-based training approach

Awareness-based training (ABT) is the traditional training approach used for security awareness [e.g., information security (INFOSEC), operational security (OPSEC)] and has become a staple component of our work environments. This type of training is typically annual, virtual, self-paced, and awareness-based (i.e., primarily driven by passive exposure to informational content)—often in the form of animated videos, slideshows, and manuals. However, despite its ease of deployment, ABT has several shortcomings including: (a) having content that is passively presented by tedious delivery formats (e.g., online slide deck); (b) is often created to fulfill a legal liability requirement and is rarely evaluated for effectiveness; (c) offers few active opportunities to apply knowledge awareness identified in the training; (d) occasionally includes post-training knowledge evaluations (i.e., tests and quizzes) that reveal poor information retention by employees; and importantly, (e) trainees are often unable to apply their learning in real-world situations after the training (Ackerman, 1992; Booth, 2007; Donkor, 2010; Tschakert and Ngamsuriyaroj, 2019; Highsmith, 2021). Such shortcomings may help explain findings in a recent study conducted by MITRE in which only 35% of employees reported to their organization after being unambiguously approached by a foreign adversary on LinkedIn (Caputo et al., 2020).

### 1.2 Skills-based training approach

There are alternatives to the traditional, awareness-based training approach. The skills-based training (SBT) approach offers that new skills are acquired through the application of awareness-based learning in simulated and real-world experiences (Kraiger et al., 1993; Vural and Bulut, 2018). From a SBT approach perspective, skill-acquisition is a process that involves information synthesis through doing (i.e., actively applying knowledge learned to novel practice situations) and receiving performance feedback on how well one is doing making the process necessarily experience-based (Dreyfus and Dreyfus, 1980; Brand et al., 2020). For example, this process is especially apparent in the acquisition of motor skills in sports contexts where simple observation of what to do (e.g., observing someone throw a ball) is not sufficient to performing well; real-world practice and performance feedback are necessary (e.g., repeatedly throwing the ball to the right place) to fully reinforce and crystalize conceptual knowledge (Kardas and O’Brien, 2018; Brand et al., 2020). SBT is thus grounded in experience which is necessary for cognitive skill development (Ackerman, 1992), and is just as prevalent in numerous work contexts outside of the domain of sports (e.g., training of air traffic controllers, pilot training, combat training, medical training, language acquisition, interpreting satellite imagery) (Cutrer et al., 2017).

In contrast to ABT, SBT has been shown to preserve and even improve learning over time as well as improve recognition of real-world situations where the learned content can be applied (Kardas and O’Brien, 2018). These benefits are due to the skills-based approach focusing on real-world simulation, practice, and subject matter expert feedback. Skills are not only knowledge, but also behaviors that can be learned and improved upon with practice. Risk recognition and reporting, by definition, are behaviors and require the application of knowledge in real-world situations. If such behaviors can be improved with training and practice, then they are skills. If the behaviors cannot be improved upon, then there is no theoretical reason why training should be conducted at all, and such training is then merely a compliance checkbox.

### 1.3 Risk recognition and reporting

Within organizations, employees can serve an integral role as human security sensors to potential security threats through appropriate recognition of risks and timely reporting (see Grispos et al., 2017). It is imperative that employees can recognize and report events that are unexpected, unplanned, not typical, and/or do not seem right (e.g., “see something, say something”). However, to achieve effective risk recognition and reporting, employees must be made aware of what security risks the organization might be facing and where to appropriately report these potential threats when they are observed, thus security awareness is key. Security awareness refers to the extent to which individuals within an organization understand the importance of security and the level of security required. For an organization, the objective of such training is to help preserve the confidentiality, integrity, and availability of information and sensitive information systems assets against attacks and threats by increasing employee’s security awareness. Security awareness deployed within information security training is designed to influence employees to behave and take actions that reduce security risks by increasing employee knowledge about risks to security and the policies and procedures of the organization to respond to such risks. Among the most common security awareness trainings include OPSEC, InfoSec, insider threat training, counterintelligence training (CI), foreign travel training, and social media training.

#### 1.3.1 Risk recognition

Recognizing security risks when they occur requires employees to be knowledgeable of what to look out for, attentive to their everyday work environments, and possess the skills to discern genuine risks from false alarms. Within a multistage intervention framework that has been reliably used to describe when bystanders intervene when witnessing a concerning event, risk recognition is a process in which individuals notice that something is occurring (or has occurred) and interpret the event as a genuine security risk (Latané and Darley, 1970). This seminal framework has been applied to a variety of contexts (e.g., crime, bullying, harassment, prosocial helping) including the understanding of insider risk and employee reporting thereof (Caputo et al., 2020). In workplace contexts, risk recognition by concerned employees is often vital to organizational security when automated cyber approaches fall short (Grispos et al., 2017). Human sensors can often observe and detect events in the work environment that seem odd or concerning in ways that cyber tools have no visibility or have yet to develop a proficiency in detection. However, for employees to be good sensors, a modicum of security training is needed to know what to look for (i.e., what constitutes an incident of concern) and when to report it (i.e., when does the observed incident cross a threshold to be reportable; Heartfield et al., 2016; Grispos et al., 2017).

#### 1.3.2 Risk reporting

Risk reporting stages of intervention involve taking responsibility for reporting, deciding how to report, and engaging in reporting behavior. A significant amount of social and behavioral sciences research has focused on reporting behavior involving the public (e.g., bystander intervention, shoplifting) or explored employee reporting in specific contexts such as safety reporting or whistleblowing. Commonly cited barriers to risk reporting often include a mix of psychological factors including individual factors such as attitudes towards reporting, knowledge of reporting process (i.e., what, when, how, and where to report), one’s role in the organization, general motivation, and error tolerance as well as organizational factors such as perceived psychological safety and management support (Pfeiffer et al., 2010). Research specifically focused on employee reporting of workplace security risks or incidents is more limited but has consistently demonstrated that employees underreport (Wood and Marshall-Mies, 2003). For instance, Wood and Marshall-Mies (2003) found that when supervisors and coworkers were engaged in suspicious behavior in a classified work environment, their colleagues underreported their observations even though reporting was mandatory. Even when employees do report security incidents, the procedure for how, when, and where employes report does not often align with official organizational policies (Grispos et al., 2017). Thus, encouraging reporting behaviors is a challenge for organizations and security trainings are typically the primary means for education promoting risk reporting.

### 1.4 Professional networking and malicious elicitations

There are numerous security domains and risk behaviors that could benefit from improving risk recognition and reporting. To adequately measure whether SBT improves employee performance in risk recognition and reporting, it is necessary that risk behaviors for training represent current risks to an organization and occur with enough frequency to be reported. One area of risk observed with increasing frequency is the use of malicious elicitation guised as benign, professional networking conversations by malign actors. Employees must regularly navigate how to safely engage in professional work activities such as networking (e.g., at conferences or external social functions) to build and maintain interpersonal relationships that benefit their careers. Networking is a particular challenge for operational security because many of the elicitation techniques used to successfully network are similar or the same as elicitation “tradecraft” used by malicious actors.

Similar in outward appearance to benign professional networking situations, malicious elicitation is *a technique that strategically uses conversation* (i.e.*, online, in writing, in person, or over the phone*) *with the sole purpose of collecting sensitive information about business operations, people, or technological assets that are sensitive and/or not publicly available*. Elicitors effectively use this technique to collect sensitive information without raising suspicion so that conversations seem non-threatening, smoothly disguised, and easily deniable if concerns are raised. When conducted by a skilled collector, elicitation will appear to be normal professional conversation, such as those that naturally occur during career networking.

There are many malicious elicitation techniques, and multiple techniques may be used in a single elicitation attempt. Through information provided by the U.S. Government online, we identified a list of common malicious elicitation techniques currently being used (see Table 1). For example, malicious elicitations include techniques where the elicitor attempts to assume knowledge about a process or technology to garner additional information or context. Another technique that may be used would be for an elicitor to provide seemingly confidential information (e.g., “Just between you and me…”). These techniques can be very effective because they are subtle and often exploit human psychological tendencies such as appealing to our desire to be polite and helpful, our desire to seem competent and knowledgeable, and/or our tendency to gossip, to name a few (see Table 1). It is important to contrast malicious elicitation techniques from everyday spear-phishing you might see in emails or text messages. Whereas spear-phishing is often focused on obtaining your personal information or installing malware via an unverified hyperlink tailoring phishes to groups of individuals, malicious elicitations are more focused on obtaining sensitive information about your work and your organization that is not widely available in public domains.

**Table 1:** Malicious elicitation technique examples and their psychological appeals.

Example elicitation techniques	Example psychological appeals
**Assumed knowledge**: pretend to have knowledge or associations in common with a person (e.g., “According to the computer network guys I used to work with…”). **Confidential bait**: pretend to divulge confidential information in hopes of receiving confidential information in return (e.g., “Just between you and me…” or “Off the record…”). **Criticism**: criticize an individual or organization in which the person has an interest in hopes the person will disclose information during a defense (e.g., “I saw on the news” or “I heard,” followed by a statement that criticizes the cleared employee’s work, company, or project). **Feigning ignorance**: pretend to be ignorant of a topic to exploit the person’s tendency to educate or teach (e.g., “I’ve had an awful time wrapping my head around…”). **Flattery**: use praise to coax a person into providing information (e.g., “I bet you were the key person in designing this new product.”). **Mutual interest**: suggest you are like a person based on shared interests, hobbies, or experiences to obtain information or build a rapport before soliciting information (e.g., “Your brother served in the Iraq war? So did mine.”)	Desire to be polite and helpful. Desire to show reciprocity. Desire to seem competent, knowledgeable, or well informed. Desire to feel appreciated and believe we are contributing to something important. Tendency to gossip. Tendency to expand on a topic when given praise or encouragement; to show off. Tendency to correct others. Tendency to underestimate the value of the information being sought or given. Tendency to believe others are honest; tendency to answer truthfully when asked an “honest” question.

Example elicitations identified by Defense Counterintelligence and Security Agency (DCSA) and Federal Bureau of Investigation (FBI).

Employees need to be able to recognize and discern what would be considered “normal professional networking” to recognize when an interaction with someone that is unexpected or unplanned is actually malicious elicitation. Therefore, the goal of the current research was to explore whether SBT is more effective than ABT at increasing an employee’s ability to distinguish between benign, professional networking and malicious elicitation behaviors.

### 1.5 An empirical study to assess effectiveness of skills-based training

To examine whether a skills-based training approach improved risk recognition and reporting of malicious elicitation messages compared to traditional, awareness-based training, an empirical study was devised. In addition, to ensure that a skills-based training approach evoked differentiation of malicious and benign messages—and not merely increased reporting across the board (i.e., an increase in false alarms)—the study also examined reporting behavior of benign, professional networking messages. Post-training, reporting behaviors were observed over a 12-month period to test for the longevity and potential rate of training decay under examination.

## 2 Method

### 2.1 Recruitment and participants

A total of 72 employees of The MITRE Corporation were recruited as participants for a “Secure and Savvy Professional Networking Training Study” (see Table 2 for sample characteristics). MITRE is a not-for-profit Defense Industrial Base organization (DIB) that operates several Federally Funded Research and Development Centers (FFRDCs). The sample size chosen was consistent with the minimum suggested number of participants for experiments with two groups. Employee participant recruitment was conducted using MITRE-wide communications mechanisms including listservs, weekly emails, internal webpages, digital message screens in common areas, and colleague networks. Recruitment materials emphasized that volunteers were sought who either have a public-facing job role, and/or self-identify as being an active networker or active in posting to online social media. However, employees from all roles and positions within MITRE were encouraged to participate. Throughout the duration of the study, three participants attritted from the study due to leaving the organization during the Testing Phase (two from ABT group and one from the SBT group). Thus, complete data was available for 69 participants with partial data utilized for the three who attrited.

**Table 2:** Summary of sample characteristics.

	Overall	Awareness-based training (ABT) group	Skills-based training (SBT) group
Sample size: *N* (%)	72 (100%)	36 (50%)	36 (50%)
Gender: *N* (%)			
*Male*	35 (51.4%)	16 (44.4%)	19 (52.8%)
*Female*	37 (48.6%)	20 (55.6%)	17 (47.2%)
Clearance level: *N* (%)			
*Uncleared*	17 (23.6%)	7 (19.4%)	10 (27.8%)
*Cleared*	55 (76.4%)	29 (80.6%)	26 (72.2%)
Job tenure in years: *M* (*SD*)	8.01 (9.36)	9.29 (10.20)	6.72 (8.37)
Attrition during the 12-Mo. study	3 (4.2%)	2 (5.6%)	1 (2.8%)

### 2.2 Materials

#### 2.2.1 Elicitation messages

A total of 40 elicitation messages were generated for the study. To ensure ecological validity, elicitations were generated based on input by insider risk subject matter experts (SMEs) from University Affiliated Research Centers (UARCs), FFRDCs, and other DIB organizations. For malicious elicitations (*N* = 30), the content in the messages was designed to reflect various combinations of elicitation techniques and red flags previously mentioned in Tables 1, 3. In addition, a set of professional networking messages (*N* = 10) were generated to act as benign distractor messages to test a participant’s discriminability between reportable malicious messages and non-reportable professional networking messages.

**Table 3:** List of red flags associated with malicious elicitations.

Concerning behavior	Example
Use of fake names or organizations	Unable to confirm the existence of entity elsewhere
Request to take a conversation “offline” or to a less secure environment	Move communication from email to a private phone number or to an encrypted messaging app (e.g., Signal)
Request sensitive or protected information	Ask for reports or information that is not released to the public
Person or event is in a location of concern	China, Russia, Iran, North Korea
Events sponsored or hosted by organizations affiliated with a location of concern	An event in Europe hosted by a Chinese organization
Feigning ignorance	Ask if you could correct their knowledge about a sensitive topic
Excessive flattery	Address you as “Excellent” or “Esteemed” scientist
Send “too good to be true” offers	Guaranteed publications; All-expense-paid travel; Excessive compensation
Requirement of foreign travel, even if not a location of concern	Any travel (for cleared employees) outside the United States (i.e., OCONUS)
Urgency	Request a speedy response without delay to avoid opportunity loss
Use of poor spelling and grammar	Writing mistakes not typical of a proficient English language speaker
Use of unknown hyperlinks	Hyperlinks that are not well-marked or may lead to malicious websites or downloads

**Figure 1:**
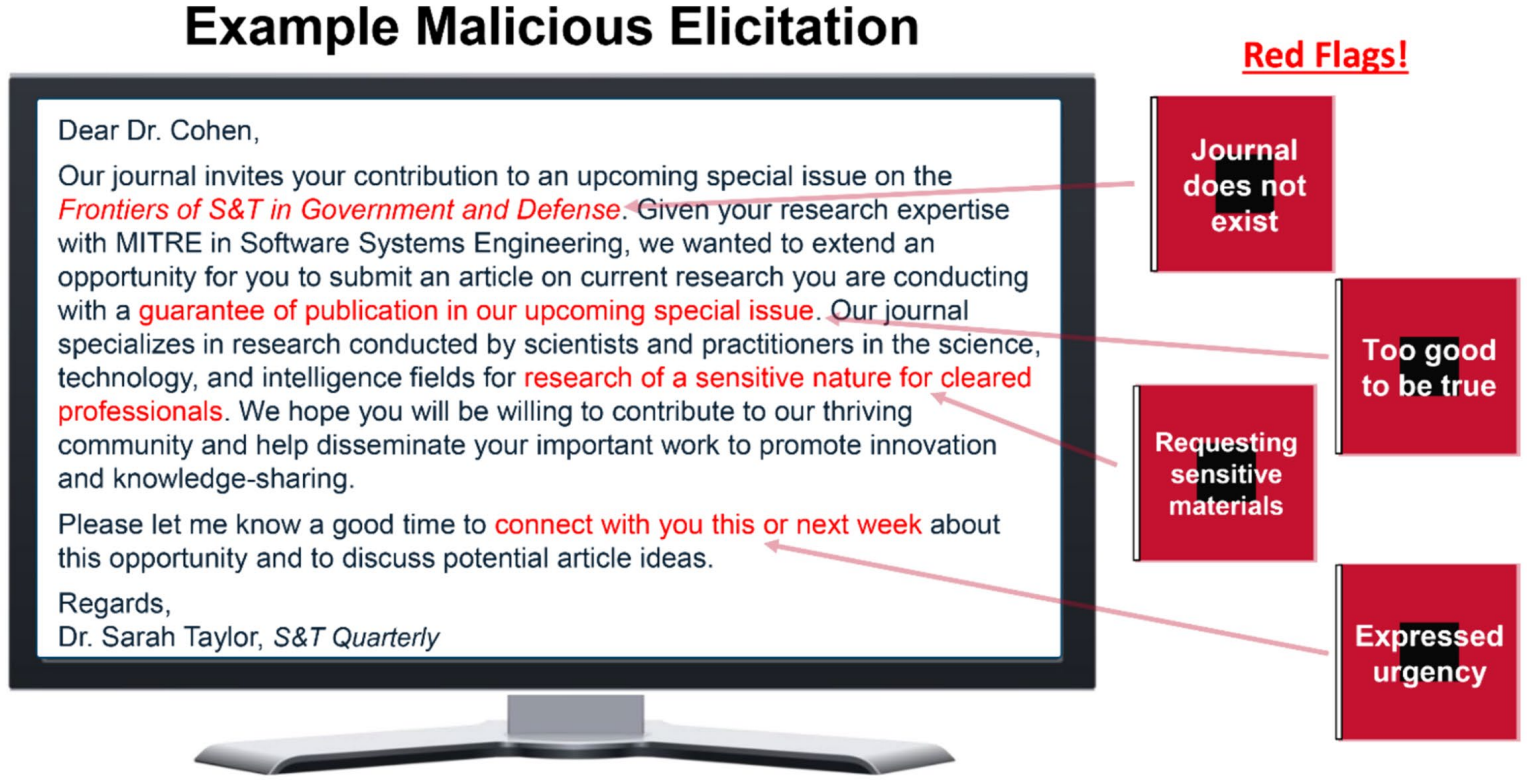
Example of malicious elicitation message with corresponding concerning behaviors or red flags.

All messages were piloted by an independent sample of 41 employee participants before the main study began. Messages were rated on the degree to which raters viewed their intent to be of a malicious or benign professional, networking nature and the perceived difficulty of discerning such intent (details available upon request). Based on the results of the pilot, a total of 26 elicitation messages were selected to be used in the study. These messages included a mixture of benign professional networking messages and malicious elicitation messages in the forms of electronic mail (i.e., e-mail) or SMS text messages with some to be used for only the SBT group during their *Practice Phase* and others to be used for all participants during the *Testing Phase* of the study (see below). A final set of malicious elicitation messages (*N* = 17) were selected from the pilot to represent messages that were unambiguously rated as possessing concerning behaviors or red flags that pointed to possible malicious intent (for an example, see Figure 1).

By contrast, a final set of professional networking messages (*N* = 9) were selected from the pilot that were of a benign intent and were included to test if participants were likely to increase their reporting behaviors on all messages they received by study proctors or if they were discerning enough to parse out messages with greater malicious intent. Benign messages used real-world organizations and links that are typical for career research and development professionals at MITRE. An example of a benign, professional networking message inviting recipients to join ResearchGate is provided in Box 1.

**BOX 1 Example professional networking message.**

**FROM**: ‘connect@researchgate.net’

**SUBJECT**: A Community of Scholars Awaits

**BODY**: If you are interested in discovering scientific knowledge and staying connected to the world of science, join ResearchGate today!

https://www.researchgate.net/

ResearchGate is a place where scholars can connect with their scientific communities to share research, collaborate with peers, network, and get the support needed to advance careers.

Join a community of 20 million scientists today and advance your research. Join for free today!

ResearchGate

© 2008–2022 ResearchGate. All rights reserved.

#### 2.2.2 Web domains and e-mail addresses

To facilitate the delivery of electronic email messages outside of MITRE systems (i.e., to obfuscate the research team’s connection to messaging), a series of nine web domains were created using the web-hosting service Bluehost. These domains were purchased with a company credit card to create the domains and subsequent email addresses associated with them. For example, the domain “frontiers-st.com” was created with associated email addresses to represent science and technology aliases. In addition to the Bluehost domains, four Google Mail (i.e., Gmail) accounts were created to represent actors posing as individuals using personal unaffiliated email accounts (e.g., a rising undergraduate senior at a nearby university).

### 2.3 Procedure

At the beginning of the study, all volunteer employee participants received a welcome email introducing them to the study and providing them with a link to an online entry questionnaire. The entry questionnaire contained the informed consent form, provided participants with instructions on how to reach the training materials for review, and a place for them submit affirmation of having reviewed the training materials. After completing the informed consent, employee participants were randomly assigned to one of two experimental groups: the awareness-based training control group (*N* = 36) or the skills-based training group (*N* = 36).

#### 2.3.1 Training phase: awareness-based training group

##### 2.3.1.1 Awareness-based training

Participants assigned to the awareness-based training (ABT) group were asked to review content related to operational security (OPSEC). These materials consisted of MITRE’s annual OPSEC training slides that cover security topics including using employee badges, access and classification types, public release policies and procedures, data spills, counterintelligence and foreign travel, insider threats & reporting, and unauthorized disclosure. Included at the end of the annual OPSEC training slides, participants were also asked to review slides covering the topic of malicious elicitation. These slides, created by the research team using some content from U.S. Government trainings as well as U.S. Government malicious elicitation guidance online, described how adversaries can disguise malicious intent to collect sensitive information using seemingly benign techniques similar to professional networking. Common concerning behaviors or red flags were presented to participants covered things like urgency, flattery, requests for sensitive information, and too good to be true offers (see Tables 1, 3). Of importance, those in the ABT group did not receive any additional training or practice. After the OPSEC and malicious elicitation trainings, ABT participants were thanked for their participation and were told that the study had concluded.

#### 2.3.2 Training and practice phase: skills-based training group

##### 2.3.2.1 Awareness-based training

Participants assigned to the skills-based training (SBT) group were asked to review the same informational training content related to OPSEC and malicious elicitation as the ABT group.

##### 2.3.2.2 Introduction to skills-based training

Following the awareness-based training content, those in the SBT group were provided a few additional slides meant to set up what to expect in the coming weeks for the skills-based training *Practice Phase*. Specifically, participants were told that in addition to the informational content they reviewed in the OPSEC training slides, they would be given the opportunity to practice the skills they learned in the awareness-based training. SBT participants were told that they would be tasked to identify and report potentially malicious elicitations that exhibit any of the concerning behaviors or red flags described in the OPSEC and malicious elicitation trainings using previously described methods of reporting (e.g., clicking on Report Suspicious Email button in Outlook, calling or emailing other security reporting processes). Participants would then receive periodic feedback (i.e., week-to-week) on how well they were applying their skills. Following feedback, SBT participants would be given more opportunities to practice again using the feedback that was provided on their individual performance. Thus, SBT was described as not only knowledge, but also behaviors that can be learned and improved upon with subsequent hands-on practice so that, in the future, participants can have the confidence to successfully apply their newly-honed skills, when necessary, during their workdays.

##### 2.3.2.3 Practice phase and feedback

Over the course of about five weeks, SBT employee participants received four malicious elicitation emails, four benign professional networking emails, and one special malicious text message sent to their personal mobile phone number (if available). Messages were spread out in such a way that, on average, they received 2–3 messages per week. For malicious messages, participants were expected to report these elicitation attempts to MITRE Global Security Services (GSS) using pre-described methods (e.g., Outlook suspicious email button, direct email or call to security). A member of GSS who was unaware of the full participant details would then inform the research team when employee reports were being made on emails with subject lines and message content summaries that had been provided previously to GSS. Feedback for SBT participants occurred periodically from week-to-week with a total of four feedback messages provided. After each practice week cycle of receiving practice messages, SBT participants were provided feedback on their individual performance at reporting potentially reportable malicious messages via email messages.^1^ Thus, participants were provided feedback on (a) whether they correctly reported or did not report a malicious elicitation message and (b) whether they incorrectly reported a benign professional networking message or correctly did not report these messages. In addition to their reporting performance, participants received detailed feedback on the content of messages sent in the previous week, their method of contact (i.e., email, text), and specific guidance on why messages were of concern or not (e.g., listing the concerning behaviors or red flags contained within a malicious email message). After the final feedback, SBT participants were thanked for their participation and were told that the study had concluded.

#### 2.3.3 Testing phase (both groups)

**Figure 2:**
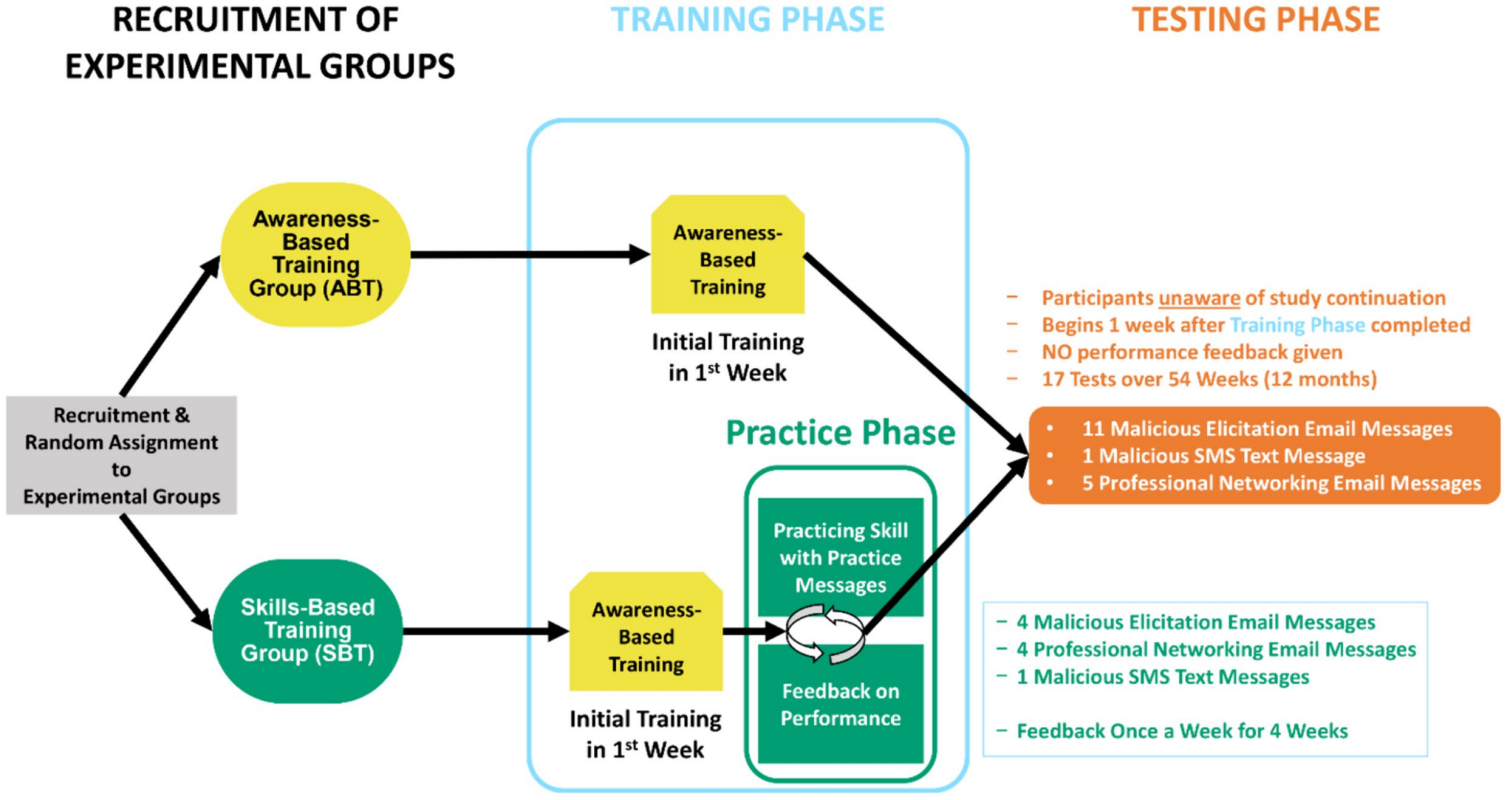
Overview of the longitudinal study design. The study design consisted of multiple phases including the participant recruitment, random assignment into experimental groups, the Training Phase, and the Testing Phase.

The *Testing Phase* was the same for participants in the ABT group and SBT group. However, the start of the Testing Phase was staggered for each experimental group to ensure that it began two weeks following the conclusion of each group’s respective Training Phase—after completion of the awareness-based training for the ABT group and after practice and feedback for the SBT group. The schedule for the testing consisted of sending 17 evaluations (11 malicious email elicitations, 1 malicious text message, 5 benign professional networking email messages) over 12 months to test risk recognition (i.e., what to report) and reporting (i.e., how to report). Participants in both groups were not given feedback on their performance at any point during the Testing Phase. In fact, following the Training Phase, both groups of employee participants believed the study to have ostensibly ended for their part and were thus unaware of testing (see Figure 2 for an overview of design and procedure). For a more detailed description of the design, materials, and procedure, see a MITRE technical report by Caputo et al. (2023).^2^

## 3 Results and findings

To test the hypothesis that skills-based training (SBT) is more effective than awareness-based training (ABT), statistical analyses were conducted on a series of focal explanatory and behavioral outcome variables. Explanatory variables included assigned *Experimental Group* (ABT vs. SBT), demographic characteristics such as *Gender* (male vs. female), *Clearance Level* (uncleared vs. cleared), *Job Tenure* (number of years at MITRE since hire date), and *Prior Reporting History* (number of reports made by participants in eight months preceding the study). Outcome variables collected during the study included message *Reporting Rates* (reported vs. did not report), message *Responding Rates* (responded vs. did not respond to messages), as well as aggregate versions of these outcome variables (i.e., percentages overall across test occasions). The following sub-sections describe the findings for the study and are broken down by Study Phase (practice, testing) and Message Type (malicious elicitation, professional networking).

### 3.1 Descriptive statistics and correlations

**Figure 3:**
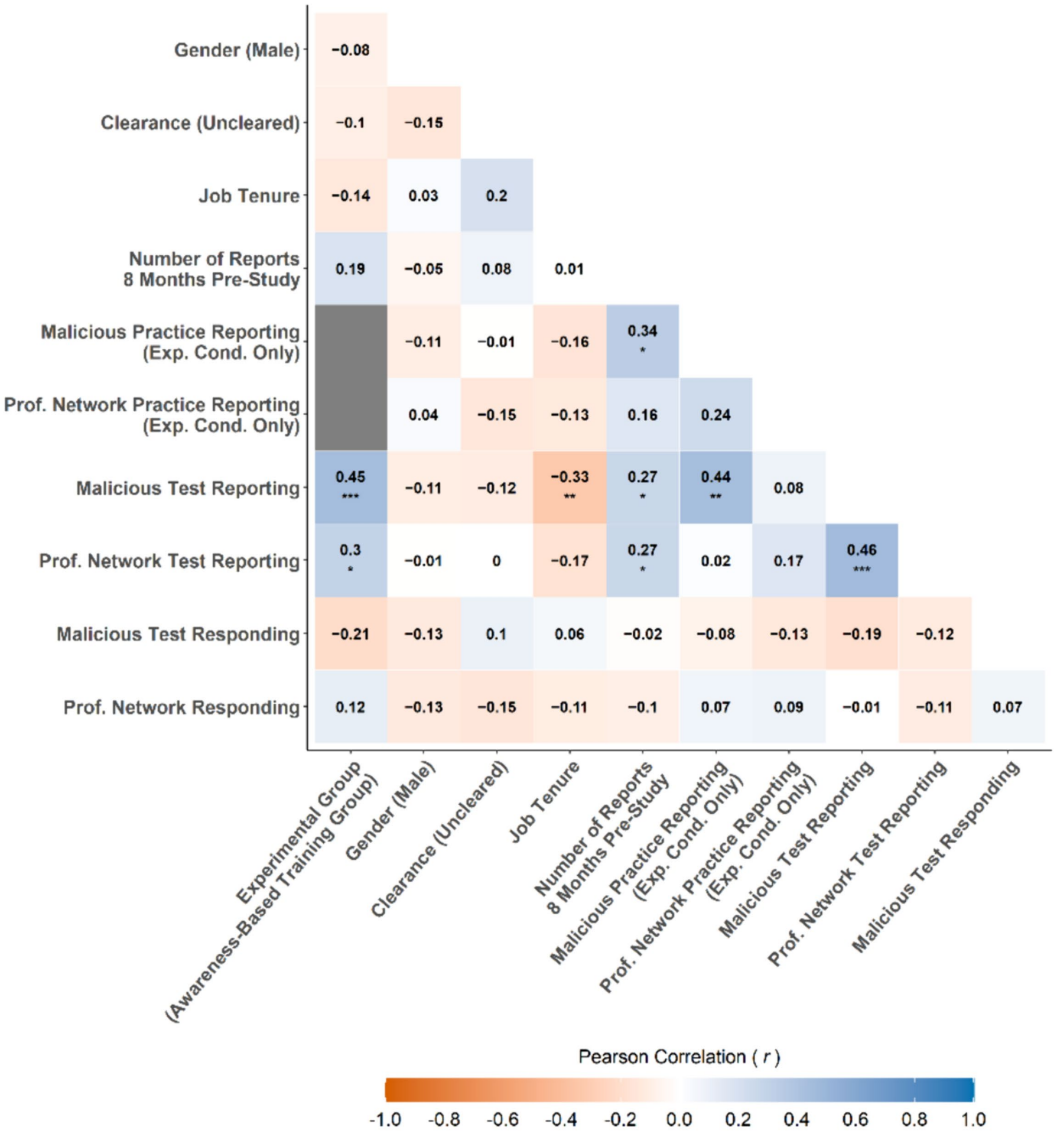
Correlation plot of focal variables across experimental groups. *Note*. For categorical variables, names in parentheses represent reference level coded as zero as used in a zero–one-indictor scheme using a point-biserial correlation method (mathematically equivalent to Pearson correlation). Correlations between continuous variables use a Pearson correlation method. *p < 0.05, ** = *p* < 0.01, *** = *p* < 0.001. Effect size: ±0.1 (small), ±0.3 (medium), ±0.5+ (Large).

To provide details on the basic statistical properties of the focal variables across both experimental groups, a summary of descriptive statistics can be found in Table 4. Similarly, a summary of zero-order correlations between focal variables can be found in Figure 3. Zero-order correlations provide the relationship between two variables without controlling for the influence of any other variables. Notable findings from analysis of descriptive statistics and zero-order correlations include:

- Participants reported twice, on average, in the eight months prior to the study. This finding indicated there was much room for improvement if the training was successful.
- Prior reporting behavior was positively related to the reporting of malicious messages in both the Practice Phase (*r* = 0.34, 95% confidence interval (CI)^3^ [0.01, 0.60], *p* = 0.045) and Testing Phase (*r* = 0.27, 95% CI [0.04, 0.47], *p* = 0.023) as well as professional networking test messages (*r* = 0.27, 95% CI [0.04, 0.47], *p* = 0.023). This finding means that participants who reported more messages before the study, reported more messages during Training and Testing Phases.
- Participants reported malicious study messages during the Testing Phase about 43% of the time, while the average reporting of professional networking study messages as malicious (i.e., a false alarm rate) was about 14%. This finding indicates that there was discernment between the message types and that, on the whole, false alarms in reporting were relatively low.
- Being in the SBT group was positively related to the reporting of malicious messages (*r* = 0.45, 95% CI [0.24, 0.61], *p* < 0.001) and, to a lesser extent, reporting professional networking messages as malicious during testing (*r* = 0.30, 95% CI [0.07, 0.50], *p* = 0.011). Thus, employees who received skills-based training reported more malicious messages than employees who only received awareness-based training.
- Gender and clearance status did not have a substantial relation with any of the variables of interest (*r*s = 0.00–0.20, *p*s = 0.991–0.085) suggesting these participant characteristics did not influence reporting or responding behavior during the study.
- Job tenure was negatively related (*r* = −0.33, 95% CI [−0.52, −0.11], *p* = 0.005) to the reporting of malicious messages during the Testing Phase. This finding meant that the longer the participant was an employee at MITRE, the less malicious test messages they reported.
- No variables had a substantial relation to responding back (via email or text message) to malicious or professional networking messages.

**Table 4:** Table of descriptive statistics across experimental groups.

Variable name	*Min*	*Max*	*Mean*	95% CI *mean*	*SD*	*Skewness*	*Kurtosis*
Prior reporting history (sum of # of reports 8 months prior to study)	0	27	2.29	[1.23, 3.35]	4.50	3.74	18.79
Malicious test reporting frequency (%)	0	81.82	42.87	[36.57, 49.12]	26.80	−0.12	1.80
Professional networking test reporting frequency (%)	0	100	14.17	[9.54, 18.80]	19.70	1.95	7.84
Malicious test responding frequency (%)	0	41.67	2.60	[1.09, 4.11]	6.43	3.74	20.69
Professional networking test responding frequency (%)	0	20	1.11	[0.03, 2.20]	4.61	3.88	16.06

### 3.2 Practice phase (SBT group only)

**Figure 4:**
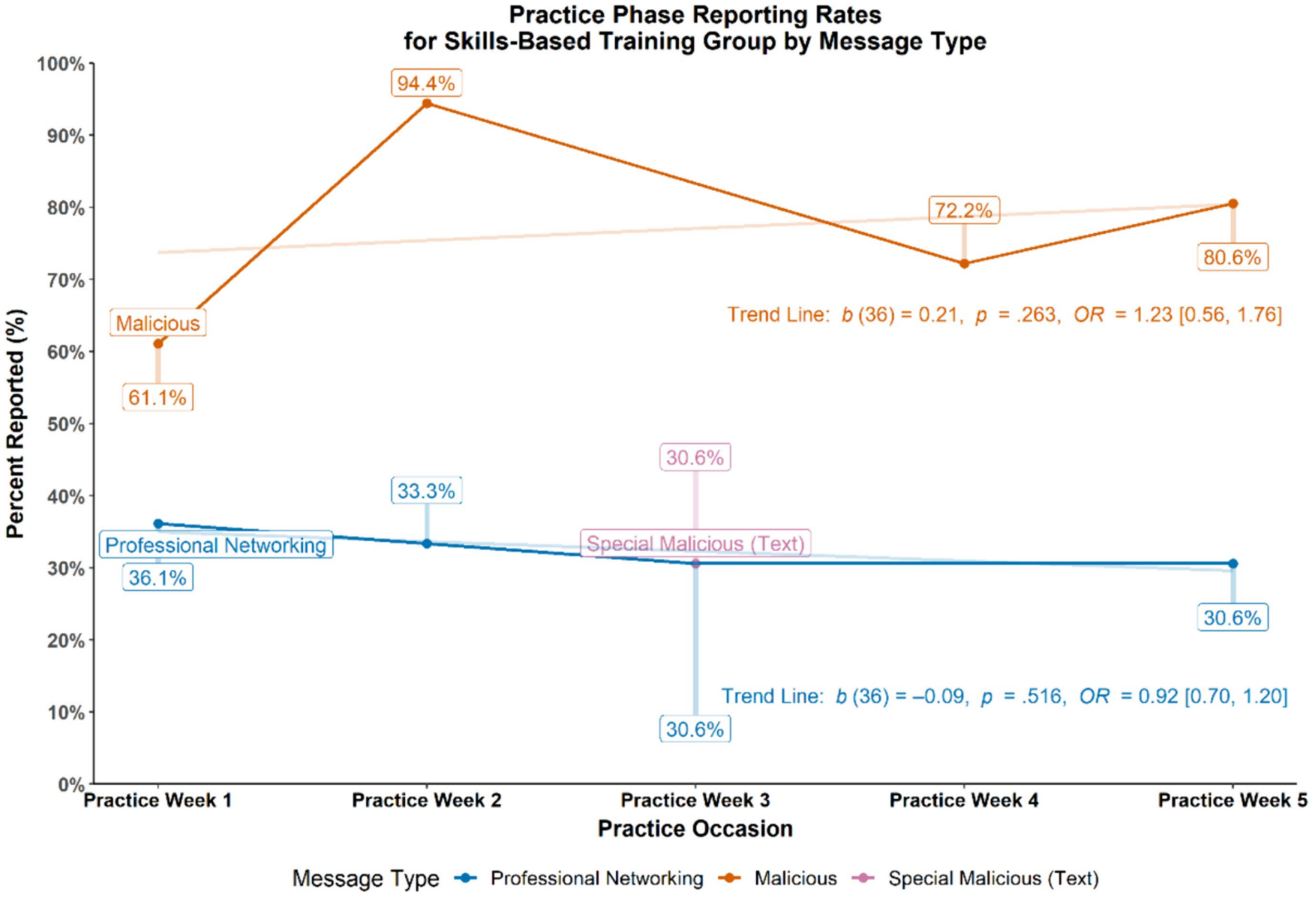
Practice phase reporting rates for skills-based training group.

For SBT participants, reporting rates were recorded for each of the practice cycles. Specifically, the percentage of those who correctly reported malicious messages (i.e., a positive elicitation detection) and the percentage of those who incorrectly reported benign, professional networking messages (i.e., a false alarm) were calculated and plotted across the five practice weeks (see Figure 4). To determine trends across time for the binary outcome of reporting or not reporting messages, a series of generalized estimating equations (GEE) were conducted. Similar to repeated measures analysis of variance, GEEs allow for testing of repeated measurements over time using dichotomous outcome data by providing estimates that indicate whether an outcome is varying from time point to time point. Results from these statistical tests revealed that SBT participants showed an increasing trend in their reporting of malicious messages (*b* = 0.21, *p* = 0.263, Odds Ratio (*OR*) = 1.23, 95% CI [0.56, 1.76]) and a decreasing trend in their reporting of benign, professional networking messages (*b* = −0.09, *p* = 0.516, *OR* = 0.92, 95% CI [0.70, 1.20]) during skills-based training. Overall, skills-based training participants reported more malicious messages (*M* = 68.8%, *SD* = 20.4%) than professional networking messages (*M* = 32.6%, *SD* = 31.5%) during the Practice Phase (*p* < 0.001, *OR* = 7.22, 95% CI [3.38, 15.20]).

To further examine discernment between malicious and benign elicitation types, the data were examined using the sensitivity index *d*′ (d prime), commonly used in Signal Detection Theory (MacMillan and Creelman, 2005). The following equation was used: *d*′ = *z*(H) – *z*(FA), where H = the hit rate (i.e., reporting malicious messages or, the signal), FA = the false alarm rate (i.e., reporting professional networking messages or, the noise), and *z* = the inverse cumulative distribution function of the Gaussian distribution. Given the unequal number of malicious (*N* = 5) and professional networking (*N* = 4) practice messages in the Practice Phase, the hit rate and false alarm rate were weighted using respective proportions out of the total of nine messages, H × 0.556 and FA × 0.444. The *d*′ index was calculated for each participant and then averaged across the SBT group to obtain the following descriptive statistics: *M*_*d*′_ = 1.01, *SD*_*d*′_ = 0.78, 95% CI [0.75, 1.28]; one-sample *t*-test (reference = “0”) *t*(35) = 7.83, *p* < 0.001, *OR* = 10.67, 95% CI [4.70, 23.76]. This result suggests that those in the SBT group differentiated malicious messages (the signal) from professional networking messages (the noise) at about 1 *SD* unit above chance.

### 3.3 Testing phase (both groups)

#### 3.3.1 Reporting rates for malicious test messages

**Figure 5:**
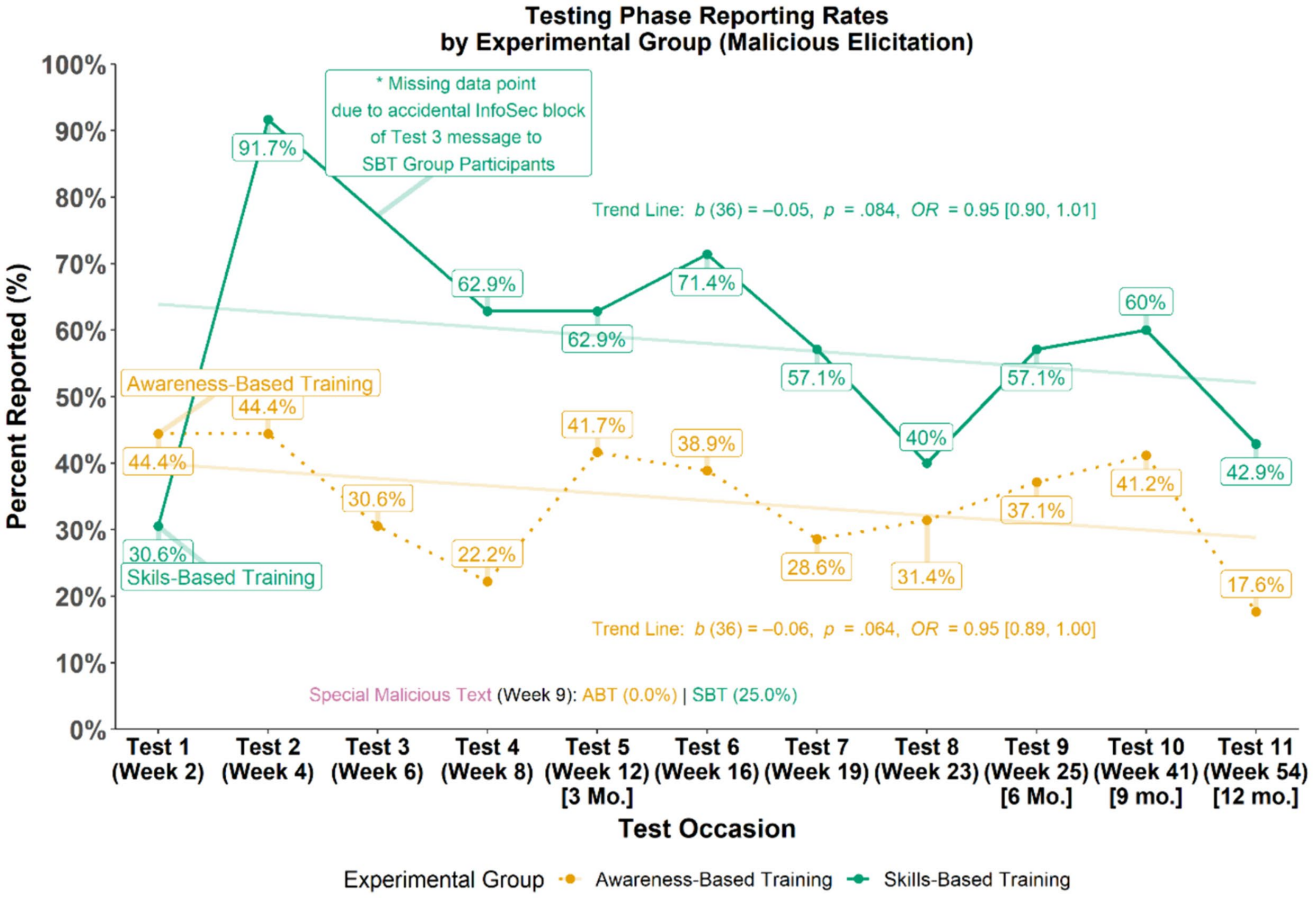
Malicious message reporting rates during testing phase.

Evaluations of employee participant reporting of malicious messages sent during the Testing Phase examined differences in the overall patterns of reporting rates (see Figure 5). GEEs were used to test if patterns in reporting rates for malicious messages varied across time (i.e., do reporting rates fluctuate between groups across time). Although the schedule for the Testing Phase was the same for all participants, participants in the SBT group were not exposed to the third malicious test messages due to it accidentally being blocked by MITRE’s Information Security.^4^ Results from the GEE analysis did not indicate any notable patterns in the change of reporting rates overall or moderated by experimental group as shown by the shallow slopes that do not intersect in the semi-transparent trend lines for each group displayed in Figure 5. However, results did reveal an overall effect of assigned experimental group (*b* = 0.95, *p* = 0.002, *OR* = 2.58, 95% CI [1.42, 4.68]) such that, *across all time points, the skills-based training group had higher reporting rates, on average, than the awareness-based training group.*

Using a series of simple logistic regressions, the magnitude of between-group differences of malicious elicitation reporting rates at each time point (i.e., test occasion) during the Testing Phase were evaluated (see Table 5). Similar to the overall pattern observed, analysis of individual test occasions revealed that those in the SBT group reported more than those in the ABT group across about 8 of 11 available test occasions with an effect size typically in the medium to large range.

**Table 5:** Malicious test reporting comparisons by experimental group.

Test occasion	Week	Awareness-based training group reporting rate [*n*]	Skills-based training group reporting rate [*n*]	Difference (%)	Effect size (*OR*)	*p*-value
Test 1	Week 2	44.4% [36]	30.6% [36]	**−13.8%**	0.55 [0.21, 1.43]	0.226
Test 2	Week 4	44.4% [36]	91.7% [36]	**+47.3%**	13.75 [4.00, 64.72]	< 0.001
Test 3	Week 6	30.6% [36]	NA [0]	NA	NA	NA
Test 4	Week 8	22.2% [36]	62.9% [35]	**+40.7%**	5.92 [2.16, 17.65]	0.001
Test 5	Week 12 (3 Mo.)	41.7% [36]	62.9% [35]	**+21.2%**	2.37 [0.92, 6.28]	0.076
Test 6	Week 16	38.9% [36]	71.4% [35]	**+32.5%**	3.93 [1.49, 10.98]	0.007
Test 7	Week 19	28.6% [35]	57.1% [35]	**+28.5%**	3.33 [1.26, 9.29]	0.018
Test 8	Week 23	31.4% [35]	40.0% [35]	**+8.6%**	1.45 [0.55, 3.95]	0.455
Test 9	Week 25 (6 Mo.)	37.1% [35]	57.1% [35]	**+20.0%**	2.26 [0.87, 6.00]	0.096
Test 10	Week 41 (9 Mo.)	41.2% [34]	60.0% [35]	**+18.8%**	2.14 [0.83, 5.71]	0.120
Test 11	Week 54 (12 Mo.)	17.6% [34]	42.9% [35]	**+25.3%**	3.50 [1.20, 11.29]	0.027
Special malicious text (text message test)	Week 9	0.0% [36]	25.0% [32]^†^	**+25.0%**	5.12 [2.03, 12.67] *	< 0.001
**Overall**	**—**	**31.0**%**[36]**	**54.7**%**[36]**	**+23.7%**	**5.93 [2.42, 14.40]**	**< 0.001**

*OR* = odds ratio. Numbers in brackets represent 95% confidence intervals. Odds ratio interpretation (Chen et al., 2010): when > 1, small (1.68), medium (3.47), large (6.71); when < 1, small (0.60), medium (0.29), large (0.15). NA, not available. † sample sizes were lower for the malicious text message test due to unavailability of personal cell phone numbers for all participants. * Due to zero reporting in one group, estimates are approximated by a likelihood ratio test of null and full models containing the experimental group variable.

Looking at the data from another angle, two multiple linear regression models were also conducted using *overall reporting rates* and *overall response rates* to malicious messages as outcome variables, respectively. The outcome variables were derived by calculating the total percentage of malicious messages reported and responded to by each participant, thus collapsing across all time points. The explanatory variables in both models included experimental group, prior reporting history, gender, clearance level, and job tenure. Results from these analyses revealed the following effects of note:

- Participants in the SBT group reported malicious test messages more often (*M* = 54.7%, *SD* = 21.1%) than those in the ABT group (*M* = 31.0%, *SD* = 26.9%; *b* = 19.11, *β* = 0.36, 95% CI [0.15, 0.57], *p* = 0.001).
- Participants in the SBT group tended to respond to fewer malicious test messages (*M* = 1.3%, *SD* = 3.9%) than the ABT group (*M* = 3.9%, *SD* = 8.1%; *b* = −2.75, *β* = −0.22, 95% CI [−0.46, 0.03], *p* = 0.085).
- Those with a prior history of reporting in the last eight months tended to report more malicious test messages during the study (*b* = 1.20, *β* = 0.20, 95% CI [−0.01, 0.41], *p* = 0.057).
- Those with longer tenures at MITRE reported fewer malicious test messages (*b* = −0.77, *β* = −0.27, 95% CI [−0.48, 0.06], *p* = 0.013).^5^

#### 3.3.2 Reporting rates for professional networking test messages

**Figure 6:**
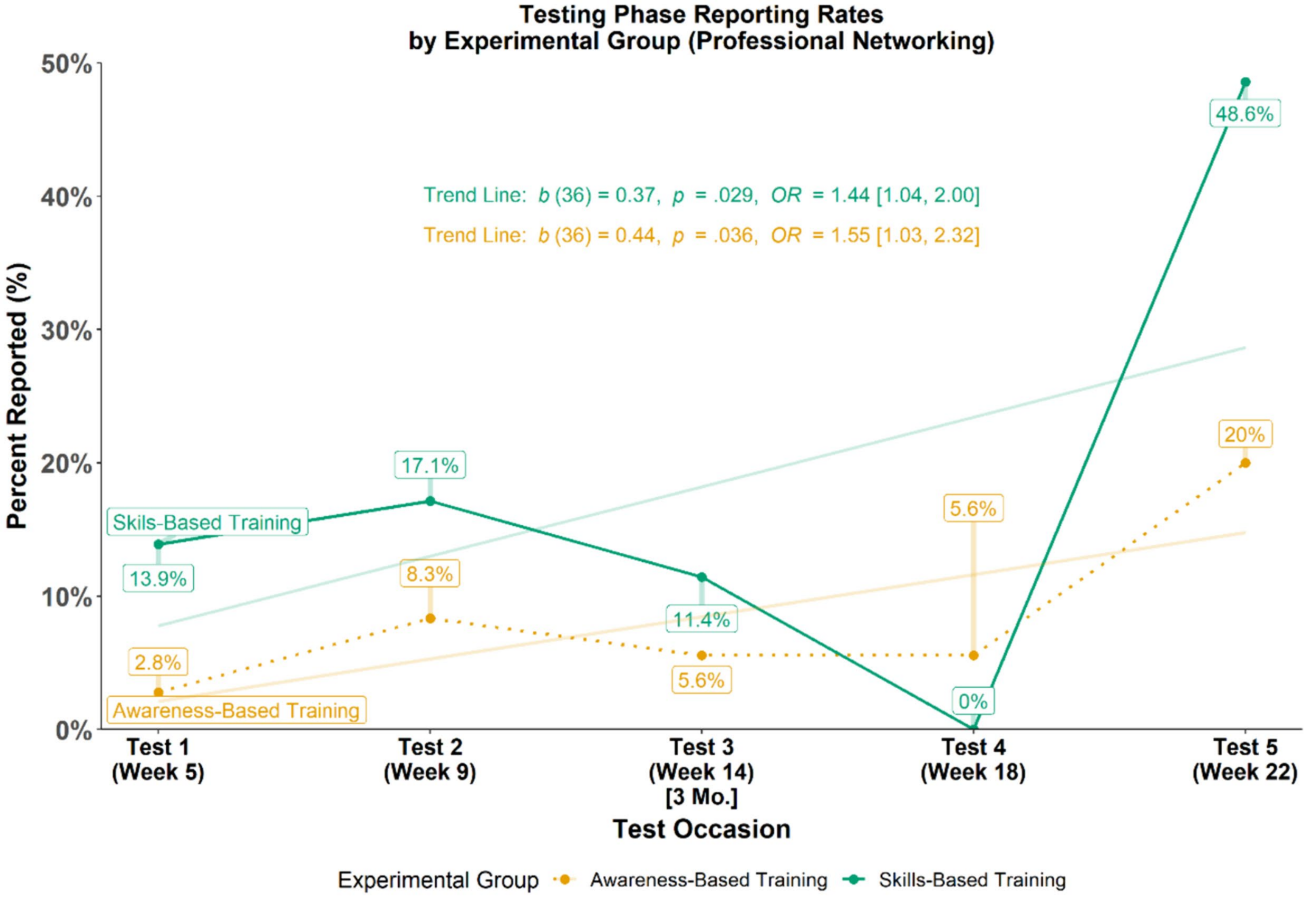
Professional networking message reporting rates during testing phase.

Similar to the examination of malicious test messages, the differences in the overall patterns of reporting rates for reporting professional networking messages as malicious, i.e., false alarm rates were examined (see Figure 6). Using GEEs, the research team tested if patterns in reporting rates for professional networking messages varied across time (i.e., do reporting rates fluctuate between groups across time). Results from this analysis revealed only an overall pattern (across groups) of reporting more professional networking messages as time passed (*b* = 0.44, *p* = 0.035, *OR* = 1.55, 95% CI [1.03, 2.32]) which is visualized in Figure 6 by the semi-transparent trend lines for each group with positive slopes that do not intersect.

Using a series of simple logistic regressions, the magnitude of between-group differences of professional networking message reporting rates at each test occasion during the Testing Phase were evaluated (see Table 6). Analysis of individual test occasions revealed that those in the SBT group reported more than those in the ABT group for only one of the testing occasions.

**Table 6:** Professional networking test reporting comparisons by experimental group.

Test Occasion	Week	Awareness-based training group reporting rate [*n*]	Skills-based training group reporting rate [*n*]	Difference (%)	Effect size (*OR*)	*p*-value
Test 1	Week 5	2.8% [36]	13.9% [36]	**+11.1%**	5.65 [0.85, 111.31]	0.123
Test 2	Week 9	8.3% [36]	17.1% [35]	**+8.8%**	2.28 [0.55, 11.55]	0.274
Test 3	Week 14 (3 Mo.)	5.6% [36]	11.4% [35]	**+5.8%**	2.19 [0.40, 16.62]	0.383
Test 4	Week 18	5.6% [36]	0.0% [35]	**−5.6%**	2.07 [0.86, 4.93] *	0.096
Test 5	Week 22	20.0% [35]	48.6% [35]	**+28.6%**	3.78 [1.35, 11.50]	0.014
**Overall**	**—**	**8.3% [36]**	**20.0% [36]**	**+11.7%**	**3.06 [1.29, 7.22]**	**0.011**

OR = odds ratio. Numbers in brackets represent 95% confidence intervals. Odds ratio interpretation (Chen et al., 2010): when > 1, small (1.68), medium (3.47), large (6.71); when < 1, small (0.60), medium (0.29), large (0.15). * Due to zero reporting in one group, estimates are approximated by a likelihood ratio test of null and full models containing the experimental condition variable.

Similar to the analysis of overall reporting and responding rates of malicious messages using multiple linear regression models (see above), the overall rates of reporting and responding for professional networking messages were also examined. Results from these analyses revealed the following effects of note:

- Participants in the SBT group reported professional networking messages more often (*M* = 18.2%, *SD* = 18.2%) than those in the ABT group (*M* = 8.5%, *SD* = 6.7%; *b* = 9.50, *β* = 0.24, 95% CI [0.01, 0.48], *p* = 0.042). Of note, this comparably higher false error rate observed for the SBT group (vs. ABT group) would be the least costly error to commit in a reporting context and is not especially high at a rate below 20%.
- Participants in both groups with a prior history of reporting in the last eight months reported more professional networking test messages as malicious during the study, indicating an effect for prior reporting history (*b* = 0.97, *β* = 0.22, 95% CI [−0.01, 0.45], *p* = 0.061).
- No effects of note were observed when examining the rates of responding to professional networking messages.

#### 3.3.3 Sensitivity between malicious and professional networking messages

To further examine discernment between malicious and benign elicitation messages, the data was examined using the sensitivity index *d*′ (d prime). Given an unequal number of malicious (*N* = 12) and professional networking (*N* = 5) messages in the Testing Phase, the hit rate and false alarm rate were weighted using their respective proportions out of the total of 17 messages, H × 0.706 and FA × 0.294. The *d*′ index was calculated for each participant and then averaged across the SBT group and the ABT group. Results of this analysis revealed that those in the SBT group had a higher sensitivity index (*M*_*d*′_ = 1.39, *SD*_*d*′_ = 0.54) compared to those in the ABT group (*M*_*d*′_ = 1.02, *SD*_*d*′_ = 0.70), *OR* = 2.95, 95% CI [1.25, 6.92], *p* = 0.014. This result suggests that, overall, those in the SBT group were relatively better at differentiating malicious messages from professional networking messages when compared to those in the ABT group.

## 4 Discussion

### 4.1 Lessons learned

Throughout the duration of the study, the researchers gleaned several lessons that can help improve the deployment of the employee skills-based training approach in future applications. One of the most significant lessons learned was that automated aspects of the feedback will make the training approach more scalable. The skills-based training approach requires an iterative cycle of practice and feedback. During the study, a human proctor manually sent emails to employees that provided feedback on their performance during training. Aspects of the process such as sending messages and employee feedback can be automated to minimize the level of effort required to execute skills-based training at scale or with large high-risk groups. Another important lesson learned was that it is important to ensure that messages are not actively being blocked by security filters. The research team worked closely with the Information Security group to ensure that methods and procedures used to send the messages would be effective, yet one message was still accidentally blocked. Therefore, it is recommended that close coordination be made with security programs to ensure that messages are whitelisted. Furthermore, it is also recommended that sending a test message to someone on the training team the day before a message is scheduled to be sent out to employees to confirm that messages will get received without issue.

### 4.2 Limitations and future directions

As an applied longitudinal research study examining employee behavior in their everyday work environments, the findings are not without a few limitations that could be addressed in future studies. First, future investigations could strive to recruit more participants than the current study (*N* = 72) to increase statistical power and to test for potential differences in additional participant characteristics like prior education/training experience, cyber hygiene habits, and general interest or motivation towards organizational security. In addition, future studies could further tease apart the potential mechanism for the observed differences in reporting between the experimental groups. For example, given the nature of the design, it could be possible that those in the SBT group spent more time reading and thinking about malicious elicitations which led them to recognize and report these messages more than the ABT group. Future examinations could better tease this mechanism apart by assigning a control group that spends an equal amount of time reviewing all of the same messages and possible feedback options that the SBT group actually practices and receives feedback on identifying and reporting such messages. Future investigations should also expand the scope of participants and security domains. For instance, future studies could examine other non-DIB organizations that might face similar security risks such as populations in the government, academia, industry, and the military. It might also be worthwhile to examine if the effectiveness of skills-based training is generalizable to security domains outside of malicious elicitation (e.g., phishing, deepfakes, physical security).

### 4.3 Conclusion

This study scientifically examined the extent to which a skills-based training approach improves real employee performance in risk recognition and reporting behaviors compared to the current awareness-based training approach. Results from this study provide empirical evidence that skills-based training can be an effective training tool to improve risk recognition and increase reporting beyond that of typical awareness-based training alone. Of note, with only five weeks of practice training—consisting of only a minimal time effort to read and report messages—employees who undertook skills-based training showed a roughly 24% improvement over traditional training that lasted for up to 12 months (see Table 5). Moreover, this finding was observed across a diverse set of employee characteristics including gender, clearance level, job tenure status, and even prior histories of reporting. Thus, skills-based training shows an effective improvement with staying power that aligns with typical annual training cycles.

Compared to annual awareness-based training, skills-based training requires employees to practice and receive feedback during training but is shown to preserve and even improve learning over time and improve recognition of real-world situations where the learned content can be applied. Findings from this empirical study indicated that five-week skills-based training module was effective to improve employees’ ability to discern and report messages that were malicious for up to 12 months post-training. Additional research is required to determine whether the training approach generalizes to other employee populations and to different content areas. Until then, researchers recommend that skills-based training be deployed annually like the typical employee training cycle for awareness-based training. Additionally, given the findings from this study, the skills-based approach would likely be appropriate for other OPSEC (i.e., reporting unauthorized disclosure caused by data spills, espionage, and/or improper safeguarding of information or the misuse of intellectual property) or insider threat areas where risk recognition is difficult to teach. Such areas could include skills-based training for foreign travel or social media use to help employees recognize other elicitation attempts.

As the first line of defense, employees need to be able to recognize and discern between what would be considered “normal professional networking” and when an interaction with someone that is unexpected or unplanned is actually a malicious elicitation seeking sensitive information. The area of *OPSEC* was selected as the risk behavior for training for this study because employees receive significant training in this area and still fail to report. This lack of reporting reduces the return on investment of currently used awareness-based training and can significantly reduce security insights into the evolution and landscape of threats aimed at an organization by malicious actors. Historically, improving employee reporting by even 20% has taken years such was the case for increasing employee reporting of spear phishing messages (Caputo et al., 2014). The findings from this study indicate that with only five weeks of skills-based training, improvements in employee risk recognition and reporting of malicious elicitation can be observed that might normally be gained with years of experience.

## Data availability statement

The raw data supporting the conclusions of this article will be made available by the authors upon request and with approval by the U.S. Government funding agency.

## Ethics statement

The studies involving humans were approved by MITRE Institutional Review Board (MIRB). The studies were conducted in accordance with the local legislation and institutional requirements. The participants provided their written informed consent to participate in this study.

## Author contributions

DC: Validation, Conceptualization, Funding acquisition, Investigation, Methodology, Project administration, Resources, Supervision, Writing – original draft, Writing – review & editing. LD: Data curation, Investigation, Methodology, Project administration, Validation, Writing – original draft, Writing – review & editing. NR: Data curation, Formal analysis, Investigation, Methodology, Validation, Visualization, Writing – original draft, Writing – review & editing.

## Footnotes

^1^We could have seen if participants opened the feedback messages but not if they fully read or understood the feedback email. However, we do know that participants continued to be engaged since, at the end of each weekly practice cycle, participants were filling out two questions asking if they received any practice messages (a survey link sent in a separate email from the feedback). Furthermore, throughout the Practice Phase, participants expected messages then feedback, so if they did not receive either they would have messaged the study’s proctor which happened once when an email did not get through the proxy.

^2^We are unable to put the intervention materials out for open access. We are currently working with existing training platforms to license the intervention into their solutions so they can be leveraged by the most organizations as a new module instead of a stand-alone training. However, the full intervention and materials used in the study can be made available to interested researchers planning a replication, please contact the corresponding author for more information.

^3^As recommended by recent guidelines on reporting of statistical tests (see Wasserstein et al., 2019) we include 95% confidence intervals for point estimates with emphasis that these values be interpreted as “compatibility intervals” that denote a range of possible values most compatible with the data under the assumed statistical model.

^4^Of note, the inclusion or exclusion of the Malicious Message Test #3 data point in analyses did not change the nature of results. For example, looking at the average reporting rates across time points with the exclusion of Test #3, participants in the SBT group (*M* = 49.8%, *SD* = 19.5%) still reported more malicious test messages than those in the ABT group (*M* = 28.5%, *SD* = 25.1%), *OR* = 5.60, 95% CI [2.29, 13.48], *p* < 0.001.

^5^We also examined the possibility of a moderated effect between experimental condition (SBT vs. ABT) and either prior history of reporting or job tenure. In both cases, there was little evidence for a meaningful effect of note. The primary effect of experimental condition with malicious elicitation reporting rates did, however, continue to hold while controlling for job tenure and prior reporting behavior in the statistical models.
